# Characterization and production of a *Bacillus mycoides* Bioflocculant for sustainable effluent treatment

**DOI:** 10.1016/j.biotno.2026.01.001

**Published:** 2026-02-07

**Authors:** Karthikeyan Harinisri, Balasubramanian Thamarai Selvi

**Affiliations:** Department of Microbiology, Sri Ramakrishna College of Arts & Science for Women, Coimbatore, 641044, Tamil Nadu, India

**Keywords:** Bioflocculant, *Bacillus* species, Exopolysaccharide, Kinetic study, Textile dyes

## Abstract

Industrial effluent treatment relies on chemical coagulants, which incur higher costs and generate toxic sludge. This study isolated and identified bioflocculant-producing *Bacillus mycoides* (S39) strain and enhanced yield using one-factor-at-a-time optimization. Characterization studies of purified bioflocculant reveal a crystalline polysaccharide bearing amine, hydroxyl, and carboxylate groups that drive adsorption and polymer bridging. The bioflocculant exhibited flocculation activities of 95.56 % for textile effluent and 92.84 % for steel wastewater with Ca^2+^ activation. Bioflocculant remained stable across pH and temperature. Kinetics followed a pseudo-first-order model, yielding rate constants, an optimized dosage, and assistance with sizing. Hemolysis (<5 %) and viability (>80 %) assays indicated non-cytotoxicity. The bioflocculant maintained performance ranged from 0.01 to 10 L, supporting S39 as a sustainable alternative to chemical coagulants. This study investigated the potential of *B*. *mycoides* bioflocculant, which showed promise as a sustainable and high-efficiency alternative to conventional coagulants and, to the best of our knowledge, the first kinetic and scale-relevant evaluation of this strain for wastewater treatment.

## Highlights

• Soil isolate *B. mycoides* (S39) confirmed by 16S rRNA sequencing method.
• The bioflocculant were purified and characterized using FTIR, SEM-EDX, and XRD analysis.
• High-performance flocculation activity while treating textile and steel industrial wastewater.
• Pseudo-first-order kinetics provide rate constants for contact-time prediction for real-world treatment.
• Robust, safe and scalable from 0.01 to 10 L.

## 1 Introduction

Access to clean water remains a critical challenge for global survival.^1^ Freshwater is essential to human life, yet its availability is scarce due to demographic expansion, urban and industrial development, and the climate crisis, fueling the water crisis. Global wastewater generation is currently estimated at 380 billion m^3^ and projected to reach 574 billion m^3^ by 2050; untreated wastewater causes health and neurological disorders.^2^ Furthermore, nutrient over-enrichment from wastewater promotes algal blooms that deplete dissolved oxygen in water bodies.^3^

During flocculation, solids in wastewater or drinking water agglomerate to form flocs, thereby clarifying the water.^4^^,^^5^ Flocculation involves the use of polymeric compounds called flocculants, which are classified into 3 categories. *Organic* and *inorganic* flocculants generate more sludge, pose environmental and health risks, and non-biodegradable disposal methods. Microbial flocculants (a type of *natural flocculant*) produced during the growth phase of microbes are referred to as bioflocculants. These extracellular polymeric substances effectively eliminate pollutants from wastewater, making it safe for environmental discharge.^4^

Numerous bacterial groups, including Actinobacteria, Bacilli, and Proteobacteria, are key producers of bioflocculant.^6^ Firmicutes, especially *Bacillus* species found in soil and applied industrially, are notable for their bioflocculant production.^7^ They also produce metabolites, antibiotics, and enzymes that benefit environmental applications.^8^ Microbial flocculants are biodegradable, eco-friendly, non-toxic, reducing sludge, secondary pollution, contaminants, and microbial load and are suitable for diverse pH and temperature conditions. They are nontoxic and pose no health risks.^9^

The research objective was to synthesize a bioflocculant derived from soil bacteria. Efficient bioflocculant-producing bacteria were identified, and optimal conditions were established to enhance their production. The bioflocculant was purified and characterized to elucidate its potential properties relevant to flocculation process. The synthesized bioflocculant was evaluated for their potential applications and kinetic properties in treating industrial wastewater.

## 2 Materials and methods

### 2.1 Isolation and identification of isolate

Aseptically collected soil samples from Coimbatore, Tamil Nadu, India, were examined. Colonies showing ropy and mucoid features were selected and inoculated into screening medium. These selected colonies were then analyzed for flocculation activity using a kaolin assay (200 rpm/min for 2 min, 120 rpm/min for 3 min, and 80 rpm/min for 10 min & settled for 10 min) and measured spectroscopically. The flocculation activity was calculated using this formula:

$$\text{Flocculation}\;\text{Activity}\;\left(FA\right)\;\left(\%\right)=\frac{\left(A-B\right)}{A}\;X\;100$$

where A and B representing control and sample measurements. The chosen colony was identified through morphological, biochemical and molecular techniques. The methodology was adapted from our previous work.^10^

Genomic DNA from isolate S39 was PCR-amplified for the 16S rRNA gene, bidirectionally sequenced with universal primers (27F/1492R), and the consensus sequences were compared through BLAST analysis at NCBI. The isolate was evolutionarily analyzed, closely matched sequences were aligned (ClustalW), and a neighbour-joining tree was built in MEGA11 using Kimura 2-parameter distances to analyse pairwise divergence. To enhance bioflocculant yield from the selected isolate, various growth parameters, including nutrient sources (Carbon and nitrogen at 1 %), pH, temperature, cation sources, inoculum volume, incubation period, and shaking speed, were optimized. The methodology was adapted from our previous work.^10^

### 2.2 Time course assay of the isolate

The production media (Sucrose – 20 g, Ammonium Sulfate – 2.5 g, KH_2_PO_4_ – 1.5 g, K_2_HPO_4_ – 4.5 g, MgSO4·7H_2_O – 0.2 g, NaCl – 0.1 g for a litre) with optimized parameters were inoculated with isolate S39 and incubated at 140 rpm for 120 h at 30 °C. At 12-h intervals, culture broth was obtained to measure culture density (OD_660_) and pH.^11^ Subsequently, 5 mL of culture broth was centrifuged for 30 min at 10,000 rpm, and flocculation activity was analyzed.

### 2.3 Production and purification of bioflocculant

A bioflocculant from isolate S39 was produced under optimized parameters. The broth was centrifuged (10,000 rpm) for 15 min at 4 °C, and supernatant was treated with twice its volume of 95 % ethanol and kept at 4 °C for 12 h to precipitate bioflocculant. Precipitate was gathered, redistributed in distilled water, and purified with a chloroform:methanol mixture (5:2 *v/v*),^7^ followed by 2-fold ethanol. After lyophilization, the purified bioflocculant was used for further studies.

### 2.4 Characterization of purified bioflocculant

This study quantified sugar content (phenol-sulfuric acid assay), with a standard (Glucose).^12^ Total protein analyzed (Lowry et al. method) with a standard (BSA).^13^ FTIR was used to examine functional groups in the 400-4000 cm^−1^ range (Shimadzu, ATR-MIRacle 10, Japan). The structural and elemental properties were characterized using SEM (Zeiss Sigma VP, Germany) and an elemental analyzer, with kaolin clay powder as the control sample. XRD technique (X'Pert Pro analytical diffractometer) was carried out using Copper Kα radiation over a scattering angle of 10–80° (2θ).^14^

### 2.5 Hemolysis assay

Bioflocculant's biocompatibility was assessed using a hemolytic assay with human blood. Blood was centrifuged at 1500 rpm for 15 min, serum removed, and red blood cells washed three times with Dulbecco's phosphate-buffered saline (DPBS). Washed cells were treated with bioflocculant (25–1000 μg/mL). Triton X-100 (0.1 %)—positive control, whereas dimethyl sulfoxide (DMSO) (0.1 %)—negative control. The control and bioflocculant solutions were incubated at 37 °C for 60 min.^15^ After incubation, the concoctions were centrifuged at 1500 rpm (5 min), and the supernatants were analyzed at 540 nm to determine the hemolytic rate (HR), which was calculated:

$$HR\left(\%\right)=\frac{\left(OD\;of\;Bioflocculant-OD\;of\;Negative\;Control\right)}{\left(OD\;of\;Positive\;Control-OD\;of\;Negative\;Control\right)}\;X\;100$$

### 2.6 Antibacterial activity of purified bioflocculant

Minimal inhibitory concentration (MIC) of purified bioflocculant was determined using broth microdilution method in 96-well plates with resazurin indicator dye. Waterborne pathogens, including *Staphylococcus aureus*, *Escherichia coli, Salmonella* sp., *Klebsiella pneumoniae*, *Enterococcus faecalis*, and *Pseudomonas aeruginosa,* were inoculated into 3 mL of Muller-Hinton (MH) broth and incubated overnight at 37 °C. The broth was then diluted to twice the volume and incubated. Bioflocculant concentrations (1–15 mg/mL) and 100 μL of each inoculum were added. The MH broth (50 μL) with each bacterial culture (50 μL) as positive control, and the MH broth as negative control.^16^ Plates were incubated overnight at 37 °C, and the MIC was determined by OD at 600 nm.

### 2.7 Antioxidant activity of purified bioflocculant

Biocompatibility and antioxidant activities of the purified bioflocculant were observed using various methods, including 2,2-Diphenyl-1-picrylhydrazyl (DPPH) scavenging activity,^16^ hydrogen peroxide (H_2_O_2_) scavenging assay,^16^ reducing power assay^17^ and ferric reducing antioxidant power (FRAP) assay.^18^ A standard stock solution of ascorbic acid (0.1 mg/mL) was prepared in ethanol. From this stock, aliquots were prepared at concentrations from 25 μg/mL to 1000 μg/mL.

### 2.8 In-vitro cytotoxicity study of purified bioflocculant

Cytotoxicity of purified bioflocculant was assessed by 3-(4,5-dimethyl-2-thiazolyl)-2,5-dimethyl-2-H-tetrazolium bromide (MTT) assay on L929 mouse fibroblast cell lines from the National Centre for Cell Science, Pune. Cells were cultured in high-glucose DMEM with 10 % FBS, 100 mg/mL of Streptomycin, and 100 units/mL of Penicillin, and maintained at 37 °C with 5 % CO_2_.^15^ Cells (2 × 10^5^) were seeded in 96-well plates with high-glucose DMEM, 10 % FBS, and 1 % antibiotic solution, incubated for 24 h at 37 °C, and washed with sterile phosphate-buffered saline. Wells were treated with bioflocculant (25–100 μg/mL) in a serum-free DMEM medium and incubated for 24–48 h. 50 μL of MTT solution was added, followed by a 4-h incubation. Supernatant was removed, wells filled with 150 μL of DMSO, and OD was measured at 570 nm, and cell viability (CV) was calculated following:

$$CV\left(\%\right)=\frac{\left(OD\;of\;Treated\;Cells\;\right)}{\left(OD\;of\;Control\;Cells\right)}\;X\;100$$

### 2.9 Effectiveness study of bioflocculant on flocculation activity

The concentration of purified bioflocculant from 0.1 to 1.0 mg/mL was assessed for flocculation activity using a kaolin assay and quantified spectroscopically at 550 nm. pH of kaolin suspension was altered between 3.0 and 12.0 by addition of 0.1 M hydrochloric acid and 0.1 M sodium hydroxide. The bioflocculant (1 mg/mL) was dissolved in distilled water, and 3 mL of this solution were heated to 20–120 °C for 30 min, followed by the evaluation of pH and thermal stability of bioflocculant by measuring the absorbance at 550 nm.^19^ A bioflocculant solution was prepared at Ca^2+^ concentrations of 0.5 %, 1 %, 2 %, and 3 %. A kaolin assay was performed by adding 3 mL of each cation concentration, and the resulting flocculation activity was measured.

### 2.10 Flocculation mechanism analysis

The zeta potential (*ζ*) plays critical role in elucidating flocculation mechanism facilitated by bioflocculant.^20^ About 0.1 g of bioflocculant was diluted, and the resulting solution was injected into a flow cell for analysis using an SZ-100 instrument (Horiba Scientific and Analytical Instruments, Japan).

### 2.11 Application of bioflocculant with industrial wastewater

The bioflocculant was applied to wastewater collected from the steel and textile industries in Tamil Nadu, India. Samples were collected, transported and preserved at 4 °C for further analysis. A bioflocculant solution (1 mg/mL) was prepared, and 3 mL of 1 % calcium chloride was added to 100 mL of each wastewater sample. Flocculation was attained by stirring mixture (200 rpm/2 min, 120 rpm/3 min, 80 rpm/10 min) followed by settling for 10 min,^21^ and upper layer was assessed for flocculation activity by measuring OD at 550 nm. Additionally, quality parameters, such as total suspended solids (TSS), biological oxygen demand (BOD), total dissolved solids (TDS), chemical oxygen demand (COD), and most probable number (MPN), were measured both before and after treatment.

### 2.12 Nutrient assessment of wastewater

Wastewater samples were collected and filtered both prior to and after treatment processes. Nutrients known to contribute to polluting waterbodies—namely nitrate, phosphorus, sodium, sulfide, chloride, and copper were subsequently analyzed. Sample extraction was performed using standardized methodologies outlined by APHA.^22^

### 2.13 Bioflocculant kinetic study with textile industry wastewater

A kinetic study quantifies removal kinetics, determines the rate constant, and optimizes dosage, making contact time predictions valuable for real-world wastewater treatment applications. To determine the optimal bioflocculant dosage, concentrations from 1 g/L to 4.5 g/L were prepared in increments of 0.5 g/L and added to 100 mL of textile wastewater.^23^ The mixtures were stirred at various speeds: 200 rpm for 120 s, 120 rpm for 180 s, and 80 rpm for 10 min, followed by settling period of 10 min. Flocculation kinetics were assessed after settling, and the bioflocculant-dye complexes were collected by centrifugation for 10 min at a speed of 4000 rpm. Dye concentrations measured by absorbance at 618 nm at time intervals spanning from 4 to 48 h.^24^^,^^25^ The dye concentration removal efficiency (RE) or removal rate was evaluated using the formula:

$$RE\left(\%\right)=\frac{\left(Co-Ct\;\right)}{\left(Co\right)}\;X\;100$$

where C_o_—the initial concentration, and C_t_—the concentration at a given time.

Three models were compared to assess the kinetic behaviour of the flocculation process: Zero-order kinetics: *C*_*t*_ = *C*_0_ – *kt*; Pseudo-first order kinetics: ln (*C*_*t*_) = ln (*C*_0_) – *kt*; Pseudo-second order kinetics: 1/*C*_*t*_ = 1/*C*_0_ + *kt*, where k was the rate constant of the respective models. Curves were overlaid on the plots to identify the best-fit line through linear regression. This curve-fitting determines the coefficient of determination (R^2^) and rate constant (slope k). R^2^ values range from 0 to 1, with highest R^2^ (>0.9) indicating the best fit across all concentrations. The optimal bioflocculant dosage was determined by maximizing the dye removal rate. Critical parameters, including Total Dissolved Solids (TDS), were also measured every 4 h over a 48-h period.

### 2.14 Flocculation activity of bioflocculant at larger volume and comparative analysis

The parameters for the production and application of bioflocculant were optimized using a kaolin suspension. The flocculation process was scaled up to larger volumes of textile wastewater, with activity evaluated at various time points (0–60 min). The volume of textile wastewater ranged from 0.01 to 10 L, and the process was conducted at ambient temperature with an effective dosage of 1 mg/mL. Conventional flocculants activity analysis of purified bioflocculant and conventional flocculants, including ferric chloride, alum, and acrylamide, was conducted using the kaolin assay method.^23^

### 2.15 Statistics and software

All experiments were conducted in triplicate. Mean and standard deviation were calculated utilizing Graph Pad Prism version 10, Origin 2024b, and Python version 3.12. Statistical significance was deduced at p values < 0.05.

## 3 Results and discussion

### 3.1 Isolation and identification of isolate

Sixty-three isolates from the soil sample were selected using Congo Red screening, from a total of 225 isolates that showed potential for exopolysaccharide production, as evidenced by black colouration on culture plates. Of these, 9 isolates displayed significant flocculation activity S14 (84.82 ± 1.89 %), S35 (70.51 ± 3.06 %), S36 (73.90 ± 2.80 %), S37 (80.55 ± 4.15 %), S39 (95.70 ± 1.04 %), S46 (69.32 ± 4.77 %), S47 (67.84 ± 1.10 %), S51 (74.19 ± 0.45 %), S62 (85.36 ± 2.45 %). The isolate (S39) was identified as an exopolysaccharide producer and showed enhanced kaolin particle aggregation in a flocculation assay. The S39 colonies displayed creamy and rhizoid features, Gram-positive rods with elongated chains, and were non-motile. The strain tested positive for catalase and could hydrolyze gelatin, starch and casein, distinguishing it from other bacteria. It has been reported that Gram-positive bacteria typically show positive results for the catalase and Voges-Proskauer (VP) tests and can also utilize carbon sources such as sucrose, glucose, and fructose, with variable utilization of xylose and arabinose.^26^

**Fig. 1:**
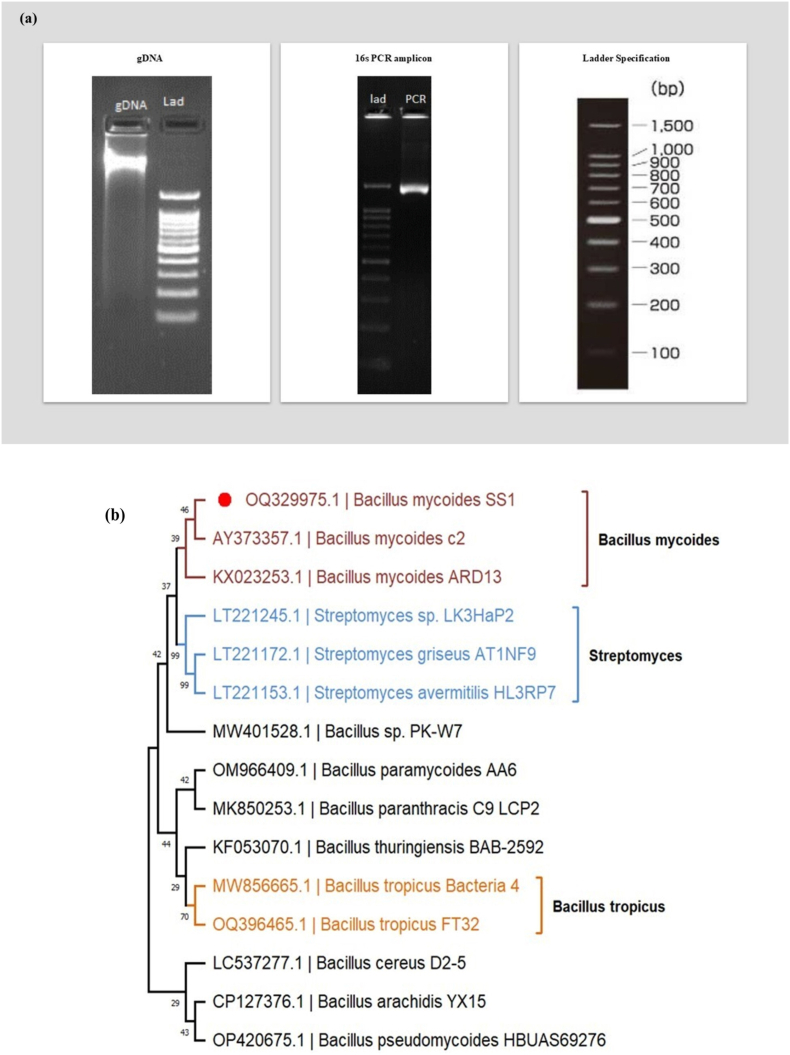
QC Data of genomic DNA isolated from S39. The agarose gel image shows a single, high-molecular weight band of strain S39 **(a)**. Construction of 16S rDNA-based phylogeny of *Bacillus mycoides* S39 (OQ329975.1) and its similar isolates. Note: Red bulletin target isolates **(b)**.

The isolate S39 revealed a similarity of 99 %, based on BLAST analysis, confirming its identification as *Bacillus mycoides* (Supplementary Excel 1). This strain, classified in the Bacillaceae family, has been deposited in GenBank under accession number OQ329975. The quality of the S39 strain data was presented in Fig. 1a. A Phylogenetic tree was created using similar sequences obtained from NCBI-BLAST (Fig. 1b), and Tajima's Neutrality Test^27^ yielded nucleotide diversity (π) value of 0.004, indicating low divergence at genus level (Supplementary Excel 2). Nucleotide composition analysis demonstrated G + C content exceeding 52 %, surpassing A + T content (Supplementary Excel 3).

**Fig. 2:**
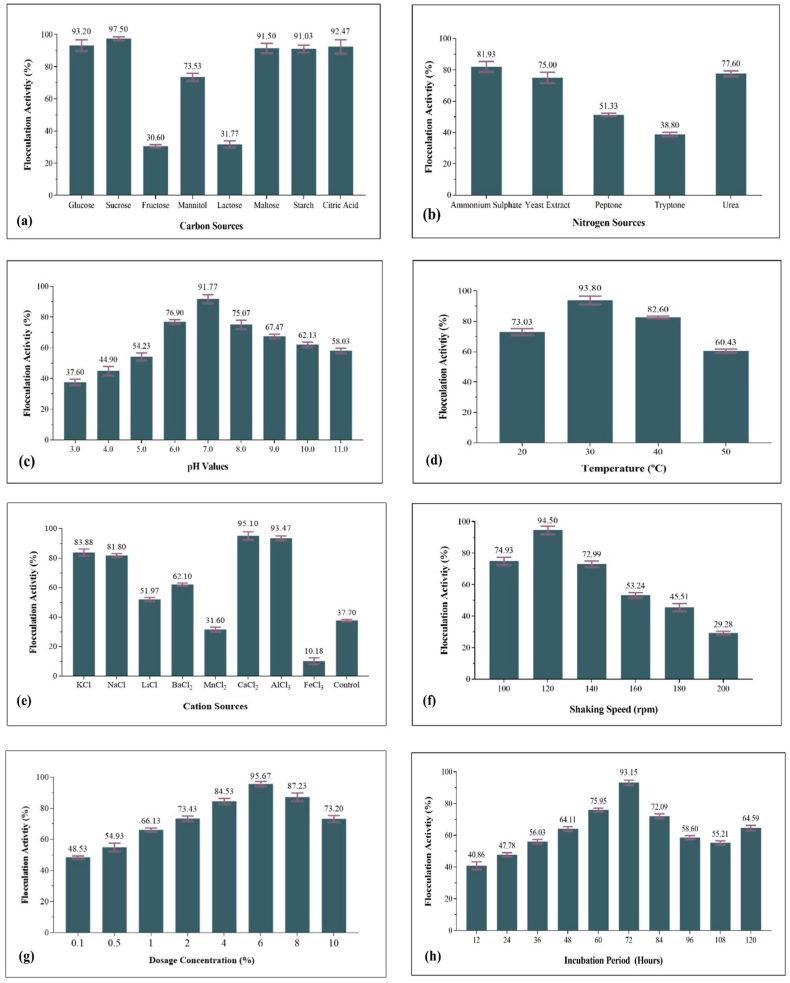
Optimization of various factors influencing flocculant efficiency in kaolin assay, including carbon **(a)**, nitrogen **(b)**, pH values **(c)**, temperature **(d)**, cation sources **(e)**, shaking speed **(f)**, dosage **(g)**, and incubation period **(h)**.

Optimal parameters for carbon (sucrose), nitrogen (ammonium sulfate), pH (7), cultivation temperature (30 °C), cations (calcium chloride), agitation speed (120 rpm), inoculum dosage (6 %), and incubation period (72 h) were identified and illustrated in Fig. 2.

### 3.2 Time course assay of the isolate

**Fig. 3:**
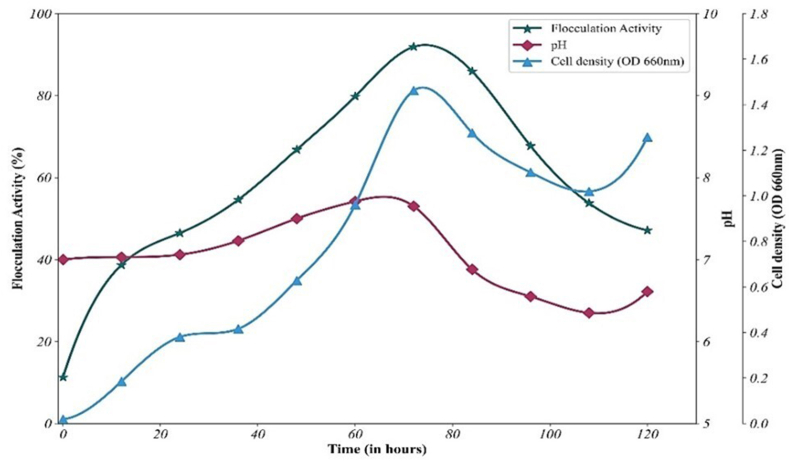
Time Course assay of *B. mycoides*.

The optimal conditions established for cultivating *B. mycoides* were utilized for the growth, which showed no cell growth during the first 12 h, indicating an early lag phase. Cell density and flocculation activity increased, peaking at 72 h with 91.8 % flocculation activity, marking the onset of stationary phase. After 84 h, flocculation activity and cell density reduced due to cell autolysis and declining enzyme activity, with the decline phase persisting until 120 h (Fig. 3). This study suggests that bioflocculant production was mainly associated with the bacterial growth phase rather than cell autolysis.^7^ Similarly, it was reported that *Pseudoalteromonas* sp. NUM8, reached the highest flocculation activity of 94.5 % at 72 h, coinciding with its stationary phase.^28^ In another study, *Bacillus salmalaya* 139SI-7 showed higher activity at 72 h, indicating the late-stationary phase.^7^

Production of bioflocculant from *B*. *mycoides* (S39) under optimized conditions yielded approximately 6.26 g/L, surpassing yields delineated by.^29^^,^^30^ In comparison, Jayaprakash et al. reported a final amount of 4.619 g/L of *B. subtilis* OL818309 purified bioflocculant.^11^ These findings demonstrate that the optimized parameters in this analysis substantially improve bioflocculant production compared to earlier studies.

### 3.3 Characterization of purified bioflocculant

#### 3.3.1 Chemical analysis of purified bioflocculant

Chemical analysis of *B. mycoides* bioflocculant revealed a total sugar content of 65.3 % and a protein content of 12.83 %. Fu et al. reported that *Pseudoalteromonas* sp. NUM8 bioflocculant was determined as a glycoprotein, consisting of 34.3 % protein and 63.4 % total carbohydrates.^28^

#### 3.3.2 Fourier transform infrared spectroscopy (FTIR) analysis

**Fig. 4:**
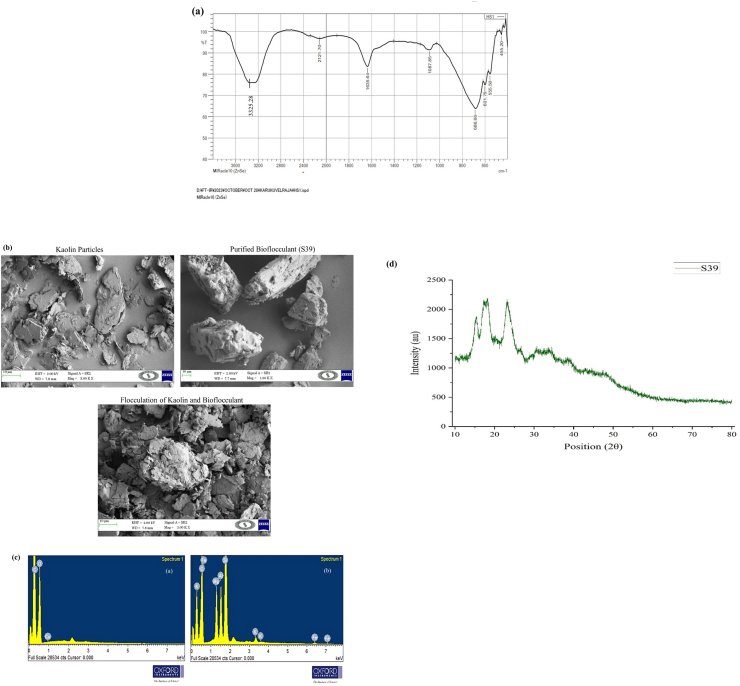
Chemical characterization of purified bioflocculant *B. mycoides* (S39) FTIR **(a)**, SEM analysis (Magnification at 10 μm) **(b)**, elemental composition **(c)**, XRD **(d)**.

FTIR spectrum of *B. mycoides* purified bioflocculant showed absorption peaks within the range of 400–4000 cm^−1^ (Fig. 4a), indicating a heterogeneous polymeric structure. Stretching band at 3325.29 cm^−1^ was the highest intensity among all peaks, suggesting the hydroxyl (O–H) groups^31^ and extensive intermolecular hydrogen bonding, a characteristic feature of polysaccharide-rich bioflocculant. Weak C–H stretching vibrations at 2700.34 cm^−1^^32^ and 2121.7 cm^−1^ correspond to aliphatic components, consistent with presence of aliphatic C–H groups.^33^ A peak at 1635.64 cm^−1^, within the amide I region, signifies the presence of C=O stretching vibrations,^34^, ^35^ confirming the presence of proteinaceous structures and associated carbonyl, carboxylic, and aromatic groups. Absorption peaks at 1087.85 cm^−1^ suggest the presence of C–O and C–*O*–C linkages, indicating polysaccharide backbone structures.^32^^,^^36^^,^^37^ Peaks at 686.66 cm^−1^ and 678.94 cm^−1^ were attributed to alkyl halide functional groups.^23^ Low-intensity peaks at 555.5 cm^−1^ and 455.2 cm^−1^ indicated presence of aryl disulfides (S–S).^35^ These were also associated with inorganic bond vibrations. The intensities and distribution of amine, hydroxyl and carboxylate moieties indicate a strong capacity for charge interactions, particle bridging, aggregation, cation adsorption and floc stability, which explains effective bioflocculant properties.

#### 3.3.3 Scanning electron microscopy (SEM) analysis

Scanning electron microscopy (SEM) analysis reveals that the bioflocculant exhibits a rough and irregular surface morphology, indicating an increased surface area for particle attachment and aggregation. SEM analysis of kaolin particles shows the presence of fine and well-dispersed particles (Fig. 4b). *B. mycoides*-derived bioflocculant displayed densely packed and uneven structural features (Fig. 4b), which enhance the ability to interact with suspended particles and promote aggregation. The rough, porous texture increases collision frequency and binding efficiency between suspended particles. Upon interaction, kaolin–bioflocculant complexes formed substantially larger and more compact flocs compared to untreated kaolin, reflecting effective aggregation and floc growth (Fig. 4b). Similar characteristics were reported for bioflocculant from *Bacillus velezensis,* which showed compact and irregular morphology leading to aggregation into larger particles.^38^ This comparative morphological corroboration supports the role of structural irregularity and surface compactness in particle aggregation efficiency and stable floc formation.

#### 3.3.4 Elemental analysis

Elemental composition of bioflocculant was analyzed to evaluate structural stability and flocculation efficacy. Presence of elements in the bioflocculant was quantified in weight percentage (% wt). The purified bioflocculant derived from *B. mycoides* contained C (Carbon) (47.96 %), O (Oxygen) (51.77 %), and Cu (Copper) (0.27 %). After flocculation, the bioflocculant exhibited a composition predominantly of O (Oxygen) (53.41 %) and Si (Silicon) (27.27 %), with minor elements including Mg (Magnesium), Al (Aluminum), Mg (Magnesium), Fe (Iron) and K (Potassium) collectively constituting 19.22 % (Fig. 4c). A previous study determined that the bioflocculant from *Bacillus subtilis* CSM5 consists of major elements: O (46.4 %), C (26.3 %) and N (5.3 %), along with minor elements P, K, Mg, Ca, S, Cl, Na, and Si.^20^ These element compositions were believed to contribute significantly to the bioflocculant's structural integrity and functional performance.^29^

#### 3.3.5 X-ray diffraction (XRD) analysis

X-ray diffraction (XRD) analysis of bioflocculant from *B. mycoides* was illustrated in Fig. 4d and was performed and recorded over a 2θ angle range of 10°–80°. The prominent 2θ peaks in the spectrum were observed between 10° and 30°, confirming its crystalline structure. These observations suggest that the bioflocculant derived from *B*. *mycoides* displayed acute peaks, highlighting its crystalline nature. Additionally, the presence of small diffraction peaks suggests the formation of smaller bioflocculant clusters and hydrogen bonds between intermolecular pairs.^39^

### 3.4 Hemolysis assay

**Fig. 5:**
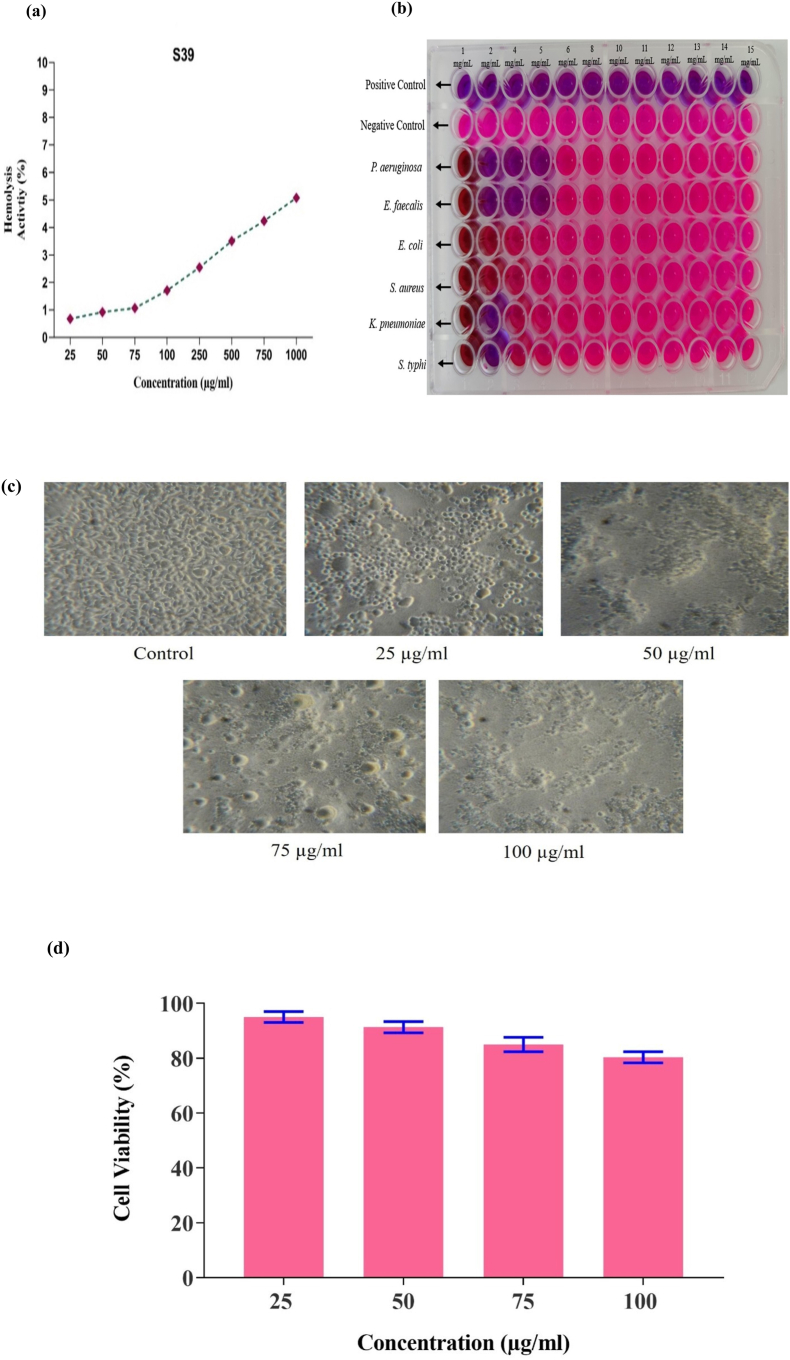
Safety Evaluation of Purified Bioflocculant *B. mycoides* Hemolysis assay **(a)**, Minimum Inhibitory Concentration (MIC) **(b)**, Cytotoxic analysis on cell lines **(c)**, Cell Viability (Data were presented in mean ± SD (n = 3) **(d)**.

The biocompatibility assessment of the bioflocculant using a hemolytic assay demonstrated minimal hemolytic activity over different concentrations of bioflocculant from 25 to 1000 μg/mL (Fig. 5a). Positive control (Triton X-100) showed complete hemolytic activity (100 %); the bioflocculant caused 0.68 % hemolysis at 25 μg/mL, increasing modestly to 5.08 % at 1000 μg/mL. This represents less disruption of red blood cells even at the highest concentration tested, suggesting a broad safety margin. Hemolysis remained low and below the accepted threshold, supporting the biocompatibility of the bioflocculant. In comparison, similarly low hemolytic activity has been reported for bioflocculant MBF-15, produced by *Paenibacillus jamilae,* with maximum hemolysis of 0.76 ± 0.14 % after 12 and 24 h of incubation.^40^

### 3.5 Antibacterial activity

This study highlights the potential of bioflocculant with antimicrobial properties as natural agents in wastewater treatment.^41^ The bioflocculant produced by *B. mycoides* exhibited significant antibacterial activity against several waterborne pathogens, with a minimum inhibitory concentration (MIC) ranging from 1 mg/mL to 15 mg/mL (Fig. 5b). Among the tested organisms, *Klebsiella pneumoniae* and *Salmonella* sp. were susceptible, showing inhibition at 5 mg/mL, whereas *Pseudomonas aeruginosa* and *Enterococcus faecalis* required 12 mg/mL for effective inhibition. *Escherichia coli* and *Staphylococcus aureus* displayed intermediate sensitivity, with inhibition at 10 mg/mL. The inhibition values quantitatively indicated differential pathogen susceptibility and support the bioflocculant potential to reduce microbial loads in wastewaters.

### 3.6 Antioxidant activity

Antioxidant activities were conducted to elucidate the mechanisms of antioxidant action, given the notable impact of free radicals on biological systems.^42^ In the *DPPH assay*, scavenging activity increased from 52.68 % at 25 μg/mL to 92.89 % at 1000 μg/mL, consistently exceeding ascorbic acid (51.65 % and 90.88 %) across all concentrations. *Hydrogen peroxide* scavenging showed lower initial activity (43.1 % at 25 μg/mL) than ascorbic acid (49.95 %) but increased markedly to 92.22 % at 1000 μg/mL, comparable to the control (91.90 %). Similarly, *reducing power assay* increased from 49.53 % at 25 μg/mL to 95 % at 1000 μg/mL, slightly surpassing ascorbic acid's concentrations (43.71 % & 94.94 %). In contrast, the *FRAP assay* increased from 35.95 % at 25 μg/mL to 73.24 ± 3.02 % at 1000 μg/mL, which was lower than the ascorbic acid control (40.25 % & 98.89 %). The results were summarized in Table 1. Overall, the bioflocculant exhibited high antioxidant capacity, particularly at elevated concentrations, indicating its ability to neutralize free radicals/oxidants present in wastewater and reduce oxidative stress in biological systems.

**Table 1:** Analysis of antioxidant activity at varying concentrations of *Bacillus mycoides* bioflocculant (25–1000 μg/mL) and ascorbic acid as standard. Results were presented in mean ± SD (n = 3).

Concentration (μg/ml)	25	50	75	100	250	500	750	1000
Standard	51.65 ± 1.22 %	62.32 ± 2.39 %	70.84 ± 1.12 %	76.17 ± 3.39 %	79.85 ± 1.79 %	82.06 ± 4.75 %	86.83 ± 2.67 %	90.88 ± 1.72 %
DPPH Assay	52.68 ± 1.16 %	64.21 ± 1.95 %	71.81 ± 3.37 %	78.80 ± 0.82 %	81.13 ± 1.51 %	83.54 ± 0.75 %	88.26 ± 1.82 %	92.89 ± 2.03 %

Standard	49.95 ± 2.67 %	54.10 ± 1.52 %	61.62 ± 2.13 %	71.50 ± 1.65 %	77.63 ± 1.09 %	82.15 ± 1.13 %	87.63 ± 2.03 %	91.90 ± 1.51 %
H_2_O_2_ Assay	43.1 ± 2.38 %	54.26 ± 2.80 %	65.39 ± 2.15 %	72.68 ± 2.54 %	78.36 ± 1.83 %	81.02 ± 1.43 %	88.11 ± 1.38 %	92.22 ± 1.74 %

Standard	43.71 ± 2.45 %	52.49 ± 1.19 %	63.52 ± 1.98 %	71.73 ± 1.60 %	76.02 ± 1.36 %	84.03 ± 2.50 %	90.75 ± 2.39 %	94.94 ± 1.40 %
Reducing Power	49.53 ± 1.63 %	54.13 ± 3.37 %	64.64 ± 2.05 %	71.99 ± 1.73 %	79.72 ± 0.74 %	85.14 ± 1.24 %	91.85 ± 1.93 %	95 ± 1.34 %

Standard	40.25 ± 1.58 %	43.42 ± 1.43 %	52.43 ± 2.04 %	72.78 ± 1.77 %	85.37 ± 1.52 %	89.24 ± 0.90 %	95.49 ± 1.43 %	98.89 ± 0.72 %
FRAP assay	35.95 ± 1,64 %	43.15 ± 1.41 %	47.35 ± 3.62 %	51.47 ± 2.16 %	57.59 ± 1.18 %	60.50 ± 0.95 %	69.11 ± 1.91 %	73.24 ± 3.02 %

### 3.7 In-vitro cytotoxicity study of purified bioflocculant

Cytotoxicity of the *B*. *mycoides* bioflocculant was evaluated using the 3-(4,5-dimethyl-2-thiazolyl)-2,5-dimethyl-2-H-tetrazolium bromide (MTT) assay, which showed high cell viability across various concentrations tested. This was shown by MTT salt turning blue-violet and forming formazan precipitates in the cytoplasm. The formazan was dissolved in DMSO, and absorbance was measured. Cell viability remained 95.33 ± 1.53 % at 25 μg/mL and 81 ± 2.65 % at 100 μg/mL, indicating non-cytotoxic behaviour at these concentrations (Fig. 5c & d). These values were comparable to literature reports: the bioflocculant from *B. subtilis* CSM5 exhibited a cell viability of 90 % at 200 μg/μL in Caco-2 cell lines,^20^ while *Pseudomonas aeruginosa* strain IASST201 showed 95.1 % viability at 10 μg and 84.2 % at 150 μg in L929 cells.^43^ The consistently high viability (>80 %) observed study supports the biocompatibility of bioflocculant and its suitability for applications involving biological exposure.

### 3.8 Effectiveness study of bioflocculant on flocculation activity

#### 3.8.1 Bioflocculant dosage influence on flocculation activity

**Fig. 6:**
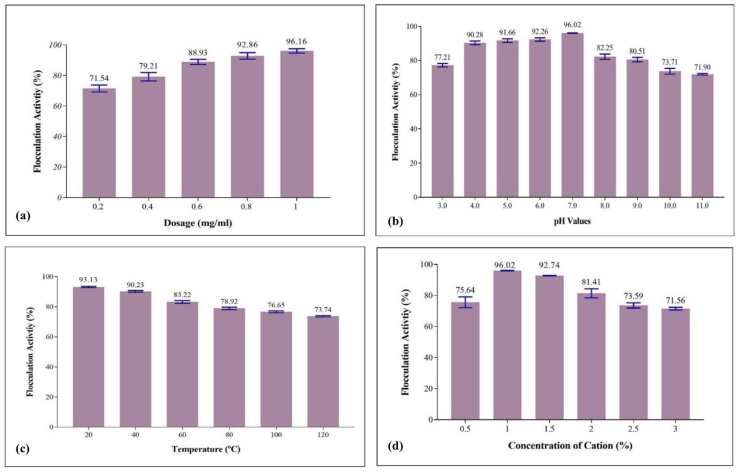
Factors influencing the flocculation activity- Bioflocculant dosage **(a)**, effect of pH **(b)**, effect of thermal stability **(c)**, cation dosage **(d)**. Data were presented in mean ± SD (n = 3) at p values < 0.05.

Purified bioflocculant from *B*. *mycoides* was examined at concentrations from 0.2 to 1 mg/mL to find the optimal dosage. Flocculation activity exceeded 70 %, peaking at 96.16 % at 1 mg/mL (Fig. 6a). Insufficient bioflocculant hampers the binding of suspended particles and increases repulsion forces between particles.^44^ Higher dosages also led to a decline in flocculation activity. Maliehe et al. reported 85.6 % activity at 0.8 mg/mL from *Alcaligenes faecalis* HCB2.^45^ Guo et al. reported 94.7 % at 12.5 mg/L with *Pseudomonas* sp. GO2.^46^

#### 3.8.2 pH effectiveness of bioflocculant on flocculation activity

pH affects bioflocculant and colloid particle charges, thereby directly influencing flocculation activity.^47^ The purified bioflocculant remained stable over pH 3.0–11.0, with flocculation exceeding 70 %, underscoring its industrial relevance and economic benefits.^48^ Maximum activity occurred between pH 4.0 and 7.0, peaking at 96.02 % at pH 7.0, with a slight decline at higher pH levels (Fig. 6b). Selepe et al. reported flocculation ranging from ∼90 % across pH 3.0–12.0 for a bioflocculant from *Ochrobactrum oryzae* AB84113, with a maximum at pH 5.0.^49^ Similarly, Maliehe et al. reported over 85 % activity from pH 5.0 to 12.0, peaking at 93 % at pH 7.0, with minor reductions at pH 3.0 to 4.0 due to reduced stability of protein components.^45^

#### 3.8.3 Thermal stability of bioflocculant on flocculation activity

The bioflocculant was tested at temperatures ranging from 20 °C to 120 °C for 30 min each, during which flocculation activity remained consistently above 70 % (Fig. 6c). At 20 °C and 40 °C, flocculation activities were 93.13 % and 90.23 %, respectively. The activity remained stable at high temperatures, with a slight decline due to protein denaturation within the bioflocculant structure. One study reported that the stability of bioflocculant from *Proteus mirabilis* at high temperatures (50–120 °C) showed maximum activity at 50 °C, decreasing at higher temperatures.^19^

#### 3.8.4 Cation dosage influence on flocculation activity

The divalent Ca^2+^ effectively enhanced flocculation by neutralizing charges of suspended particles and residual charges on bioflocculant.^50^ Ca^2+^ concentration ranged from 0.5 % to 3 %, with activity exceeding 70 %; maximum at 1 % achieving 96.02 % (Fig. 6d). *B. mycoides* bioflocculant was a cation-dependent bioflocculant, and calcium chloride was used in subsequent analyses. The bioflocculant from *Proteus mirabilis* AB 932526.1 was reported to be effective with Mg^2+^ and Mn^2+^, yielding 98 % activity;^19^ other cations exceeded 60 %, likely due to increased surface area. Another study reported that adding 10 % Ca^2+^ increased the activity of *Bacillus* spp. UPMB13 bioflocculant to 86.8 %.^50^ Ca^2+^ stabilizes negative charges on functional groups, aiding floc formation.^47^

### 3.9 Flocculation mechanism analysis

**Fig. 7:**
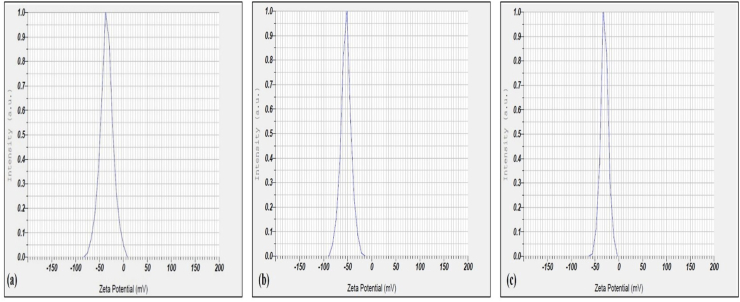
Zeta potential analysis of Bioflocculant produced by *Bacillus mycoides***(a)**, Kaolin suspension with Ca^2+^ addition – before flocculation **(b)** Addition of bioflocculant with kaolin suspension Ca^2+^ addition – after flocculation **(c)**.

**Fig. 8:**
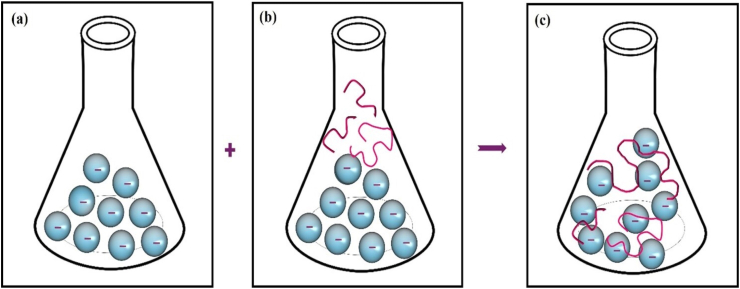
Proposed flocculation mechanism: Kaolin suspension **(a)**, Addition of bioflocculant produced by *Bacillus mycoides***(b)**, Formation of flocs due to the bridging mechanism **(c)**.

Zeta potential (*ζ*) measures electric potential at particle surfaces relative to the surrounding liquid medium. The zeta potential of a kaolin suspension in the presence of Ca^2+^ was −35.3 mV; purified bioflocculant was −30.2 mV. After bioflocculant addition, the zeta potential was −53.5 mV (Fig. 7a, b, and 7c). Zeta potential after bioflocculant addition suggested a predominant polymer-bridging mechanism,^51^^,^^52^ driven by polysaccharides that form complex structures by bridging kaolin particles.^23^ Functional groups in the bioflocculant, such as polysaccharide and hydroxyl groups, enhanced flocculation (Fig. 8). The bridging mechanism links contaminants into stronger flocs, depending on bioflocculant structure, functional groups, and molecular weight.^53^, ^54^ A bridging mechanism was identified for a bioflocculant from *Bacillus subtilis* CSM5, with a ζ of −16.5 ± 1.1 mV, and that, after flocculation in the presence of Ba^2+^, it was −5.5 ± 2.1 mV.^20^ The zeta potential of *Enterobacter* sp. ETH-2 bioflocculant was recorded at −28.7 ± 8.23 mV, while clay suspension was at −35.6 ± 1.66 mV.^53^ Kaolin suspension with ETH-2 was found to be −46.9 ± 6.72 mV.

### 3.10 Application of bioflocculant with industrial wastewater

**Fig. 9:**
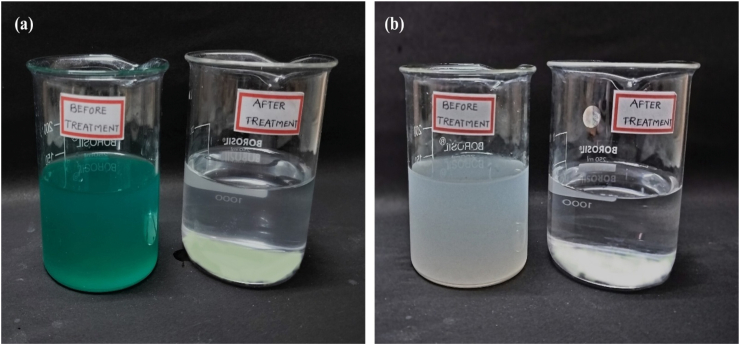
Effectiveness of *Bacillus mycoides* bioflocculant in flocculation by kaolin assay for textile industry wastewater **(a)**, steel industry wastewater **(b)**.

Wastewater from textile and steel industries is polluted with heavy metals, toxic organics, dyes, solids, and extreme pH. Textile wastewater, rich in dyes and chemicals, is challenging to treat; therefore, the effectiveness of *B. mycoides* bioflocculant was evaluated. Flocculation activity was 95.56 % for textile wastewater and 92.84 % for steel industry wastewater (Fig. 9a & b). Flocculation removed about 96 % of total dissolved solids from textile and 90 % from steel wastewater. BOD, COD, and TSS were reduced by 75 %, 71 %, and 76 % for textile wastewater and by 75 %, 68 %, and 54 % for steel-industrial wastewater, respectively. Nutrients, which play a key role in water quality standards,^55^ like sodium, sulphide, phosphorous, copper and chloride reduced by 72 %, 76 %, 94 %, 76 % and 93 % in textile and 73 %, 70 %, 94 %, 74 % and 81 % in steel wastewater, respectively (Table 2). The microbial load in both wastewater samples was reduced by 90 % following treatment. It was reported that *Bacillus subtilis* 35A bioflocculant achieved 95 % dye decolourization, effectively removed heavy metals Cr^6+^ (41.05 %) and Cu^2+^ (48.93 %) and reduced ammonia nitrogen, COD, total nitrogen, and phosphorus by 26.87 %, 51.16 %, 37.76 %, and 55.81 %, respectively.^56^ Agunbiade et al. found that *Bacillus velezensis* bioflocculant reduced brewery wastewater turbidity by 72 %, with reductions in COD by 62 % and BOD by 53.6 %, respectively.^38^

**Table 2:** Physicochemical, biological and nutrient parameters of industrial wastewater treated before and after treatment with *Bacillus mycoides* bioflocculant.

Physical Parameter	Textile Effluent	Treated Textile Effluent	Industrial Effluent	Treated Industrial Effluent
pH	9.47	7.29	8.2	7.6
Colour	Dark Green	Pale green	Milky White	Colourless
Odour	Objectionable	Agreeable	Objectionable	Agreeable
TDS (ppm)	6121	1089	1380	138
EC (μs/cm)	12,303	3698	763	295
Salinity %	0.68	0.18	0.04	0.004
Resistivity (KΩ)	2.205	5.325	19.92	13.37
ORP (mV)	−155	−254	38	19
BOD	309.47	77.15	553.12	138.72
COD	821.92	237.47	528.84	170.44
TSS	289.20	69.00	342.16	158.11
Flocculating Activity %	–	95.56 %	–	92.84 %
Microbial Log Reduction	189 × 10^4^	1-log reduction (90 %)	692 × 10^4^	1-log reduction (90 %)
Sodium (mg/L)	29.60	8.29	38.50	10.18
Sulphide (mg/L)	38.27	9.02	43.64	12.97
Nitrate (mg/L)	89.01	12.45	94.62	28.49
Total Phosphorous (mg/L)	91.16	4.68	142.55	8.53
Chloride (mg/L)	36.58	2.55	48.49	6.15
Copper (mg/L)	8.23	2.01	15.80	4.06

In this study, industrial wastewater treated with the bioflocculant *B*. *mycoides* exhibited significant reductions in parameters, enabling reuse in multiple applications, including potable and non-potable uses such as industrial reuse, agricultural irrigation, urban applications, and cleaning purposes.^57^

### 3.11 Bioflocculant kinetic study with textile industry wastewater

The flocculation efficiency of the *B. mycoides* bioflocculant demonstrated sustained effectiveness when applied to textile wastewater. To quantify dye concentrations, optical density was measured at 618 nm at 4-h intervals over 48 h. The results showed a progressive decrease in dye concentration over time across all bioflocculant concentrations (Table 3).

**Table 3:** The removal efficiency of dye from the textile wastewater was expressed in percentage (%).

Time (hours)	Bioflocculant concentration (mg/mL)							
	1	1.5	2	2.5	3	3.5	4	4.5
0	19.95	20.51	19.88	25.02	25.47	25.29	24.66	24.07
4	21.78	22.13	24.28	26.25	55.53	50.44	25.09	23.85
8	25.49	25.88	28.25	29.16	63.03	53.36	30.33	27.23
12	28.34	29.90	32.51	37.83	68.38	59.88	38.77	33.38
16	30.40	33.37	38.59	41.83	76.12	66.32	42.92	39.07
20	33.40	38.34	41.03	45.57	79.80	69.25	46.88	43.67
24	35.43	40.60	43.44	51.12	87.35	70.39	51.68	48.49
28	37.63	43.11	49.27	56.43	90.38	70.74	57.56	51.31
32	39.39	46.21	59.10	58.58	92.51	75.29	59.48	57.49
36	41.89	28.25	61.18	59.80	93.09	81.42	60.63	60.80
40	46.69	55.69	65.56	64.92	91.19	84.30	65.91	66.47
44	48.98	59.40	72.46	72.59	94.24	86.62	74.05	78.19
48	50.76	64.96	78.40	80.16	94.30	87.68	80.73	67.58

We fitted three models of different orders to the dye-removal data by least-squares regression. The validity of the chosen model was evaluated using the regression coefficient (R^2^) and root mean square error (RMSE), with thresholds defined across all bioflocculant concentrations considered for validation (R^2^ > 0.9 and RMSE <0.1). The rate constant (k) quantifies the reaction rate for dye removal and was calculated from the slope of the linear equation. The concentration corresponding to the highest k value was considered the optimal dosage for dye removal efficiency.

**Fig. 10:**
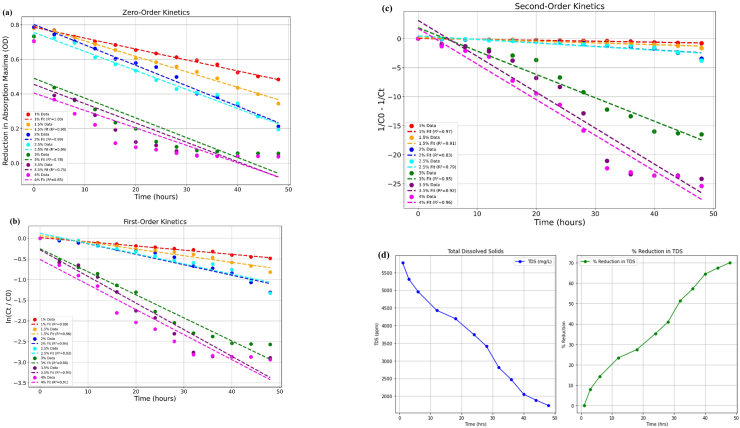
The dye removal efficiency of bioflocculant produced by *Bacillus mycoides* represented as **(a)** Zero order, **(b)** Pseudo-first order, **(c)** Pseudo-second order kinetic graph, Total Dissolved Solids (TDS) from textile wastewater **(d)**.

The zero-order kinetics, described by the equation: $C_{t}=C_{0}-\text{kt,}$ posits that the rate of dye removal was independent of its concentration. Plotting $C_{t}$ vs t (Fig. 10a) showed $R^{2}$ < 0.9 for and RSME >0.1 nm for 3 and 3.5 mg/mL bioflocculant doses, indicating a poor fit (Table 4). Pseudo-first-order kinetic model, represented by $\ln\left(C_{t}\right)=\ln\left(C_{0}\right)-\text{kt}$, indicates that rate of dye removal was directly proportional to concentration of dye present. Analysis of the concentration of dye removal $\ln\left(C_{t}/C_{0}\right)$ when plotted against time (t), the data reveal a consistent linear trend across various bioflocculant concentrations (Fig. 10b). $R^{2}$ > 0.9 and an RMSE <0.1 nm was observed for all bioflocculant doses (Table 4). The pseudo-first-order model adequately described the dye removal process by the bioflocculant in textile wastewater treatment, as indicated by high correlation coefficients (R^2^ > 0.9) across all concentrations and the lowest mean RMSE (0.061) and MAE (0.052). The rate constant (k) derived from the graph closely matches the calculated rate constant, further supporting the model. The pseudo-second-order model, given equation $\frac{1}{C_{t}}=\frac{1}{C_{0}}+\text{kt}$ suggests that the removal of dye was directly proportional to square of dye concentration. $\frac{1}{C_{t}}-\frac{1}{C_{0}\;}$ vs t plot displays deviation from linearity ($R^{2}<0.9)$ and high error RMSE >0.1 nm for several concentrations of bioflocculants (Fig. 10c) (Table 4). The pseudo-first-order model best described the dye removal process, with the highest reaction rate observed at a bioflocculant dosage of 3 mg/mL (k = −0.056), while increasing the dosage did not improve the removal rate (Table 4). Total dissolved solids (TDS) were a crucial parameter in assessing water quality. The removal of TDS was a critical consideration in wastewater treatment processes.^58^ Initially, a reduction of Total Dissolved Solids (TDS) was recorded at 8.01 %. Over time, the effectiveness of TDS removal improved significantly, reaching 70 %. The optimal dosage effectively reduced dye concentration and positively influenced physicochemical parameters, including total dissolved solids (TDS) (Fig. 10d).

**Table 4:** Zero, Pseudo-first order and pseudo-second order kinetics parameters.

Bioflocculant Dose (mg/mL)	R2 (from graph)	K (from graph)	K (calculated)	RMSE (nm)	MAE (nm)
*Zero Order Kinetics*					
1	1	−0.00632	−0.00630	0.0066	0.0053
1.5	0.99	−0.00895	−0.00909	0.0194	0.0167
2	0.99	−0.01182	−0.01197	0.0232	0.0173
2.5	0.99	−0.011	−0.01128	0.0278	0.02144
3	0.78	−0.01144	−0.01408	0.2578	0.2414
3.5	0.87	−0.01029	−0.01276	0.1799	0.1701
4	0.99	−0.01124	−0.01147	0.0249	0.0178
4.5	0.98	−0.012	−0.01139	0.0510	0.0461
*Pseudo-First-Order Kinetics*					
1	0.99	−0.01011	−0.01013	0.0142	0.0112
1.5	0.96	−0.01615	−0.01707	0.0428	0.0384
2	0.94	−0.02539	−0.02731	0.0725	0.0620
2.5	0.93	−0.02465	−0.0277	0.0661	0.0604
3	0.96	−0.0558	−0.05355	0.0651	0.0510
3.5	0.97	−0.03392	−0.03755	0.0691	0.0525
4	0.94	−0.02552	−0.02841	0.0635	0.0578
4.5	0.91	−0.02712	−0.02756	0.0954	0.0839
*Pseudo-Second-Order Kinetics*					
1	0.97	−0.01642	−0.01013	0.0252	0.0210
1.5	0.91	−0.03033	−0.01707	0.0660	0.0590
2	0.83	−0.06026	−0.02731	0.1202	0.1035
2.5	0.79	−0.06147	−0.0277	0.1090	0.0980
3	0.95	−0.40243	−0.05355	0.0790	0.0509
3.5	0.91	−0.13404	−0.03755	0.0486	0.0396
4	0.8	−0.06489	−0.02841	0.1088	0.0976
4.5	0.79	−0.06911	−0.02756	0.1416	0.1235

Similarly, Bisht et al. investigated the kinetics of COD and the decolourization of dyes,^23^ while Muthulakshmi et al. examined the adsorption kinetics pertaining to specific cationic dyes.^24^

### 3.12 Flocculation activity of bioflocculant at a larger volume and comparative analysis

**Fig. 11:**
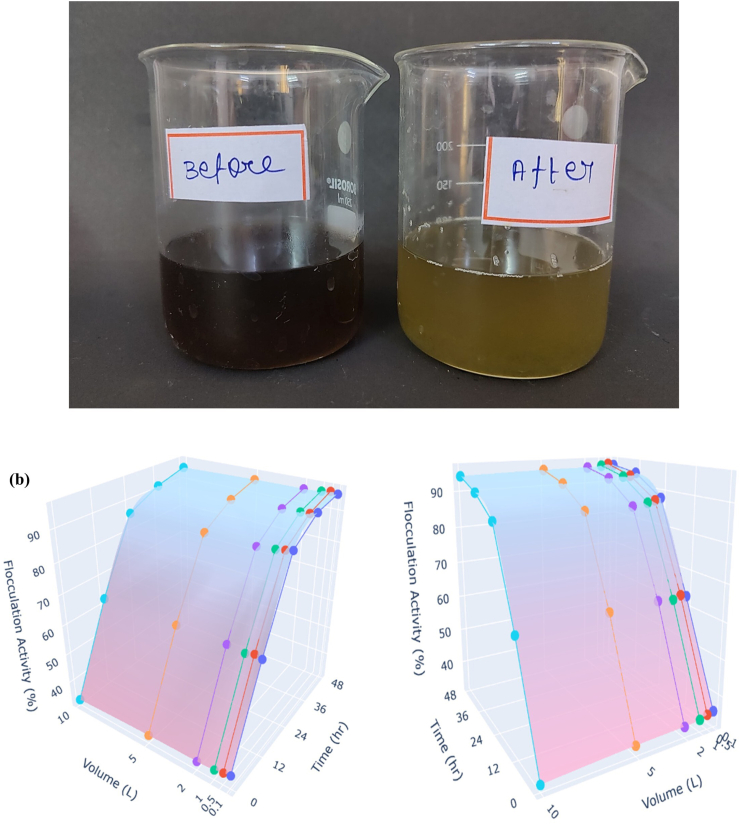
Larger-volume treatment of textile wastewater using *Bacillus mycoides* bioflocculant. Before and After flocculation **(a)**, flocculation activity **(b)**.

As a significant advancement in large-scale wastewater treatment, a feasibility study was accomplished on textile wastewater treated with bioflocculant from *B*. *mycoides* (Fig. 11a). pH of the textile wastewater remained constant, and an optimized dosage of 3 mg/mL was added along with Ca^2+^. Thorough and uniform mixing was performed to ensure an even distribution of the bioflocculant throughout the wastewater. Flocculation performance at 0.01 L was highest at 10 min, with longer reaction times resulting in decreased flocculation activity. For volumes of 0.1 L, 0.5 L, 1 L, 2 L, 5 L and 10 L, flocculation activity showed stability and increased flocculation activity at 48 h, with the flocculation activity exceeding 90 % (Fig. 11b). This stability and increased flocculation were observed during treatment with larger volumes, resulting in the formation of significant flocs. The findings suggest a promising opportunity for industrial-scale wastewater treatment, applicable to a wide range of wastewater treatment processes.

Among these, the bioflocculant derived from *B*. *mycoides* demonstrated the highest flocculation activity, achieving 96.37 ± 1.80 %. In comparison, conventional flocculants, such as ferric chloride, exhibited activities of 43.45 ± 1.31 %, alum 68.20 ± 1.75 %, and polyacrylamide 71.67 ± 2.08 %. This study highlights the potential of the *B*. *mycoides* bioflocculant to exhibit superior efficacy in treating synthetic wastewater containing kaolin.

## 4 Conclusion

The soil bacterium *Bacillus mycoides* (S39) was isolated and exhibited a high G + C content and genetic stability. Under optimized conditions, S39 produced a polysaccharide-rich bioflocculant with functional groups that enhance adsorption and polymer bridging. Characterization studies like SEM and XRD confirmed its crystalline structure. Safety assays confirmed non-hemolytic and non-cytotoxic properties (cell viability >80 %) and antibacterial and antioxidant activities. Optimal 1 mg/mL dose, especially with Ca^2+^ (1 %) addition, demonstrating effective treatment outcomes and cost efficiency. The bioflocculant effectively treats acidic and alkaline wastewater (with a peak at neutral pH) and was thermally stable over a wide range (20–120 °C), with bridging mechanisms underpinning larger floc formation. The bioflocculant achieved 95.56 % and 92.84 % flocculation efficiencies for textile and steel wastewater, respectively, with marked reductions in TDS, BOD, COD, TSS, pH, colour, and odour, and a 1-log (90 %) reduction in microbial count. Nutrients linked to eutrophication, like sodium, sulphide, nitrate, phosphorus, chloride, and copper, were also significantly reduced. Pseudo-first-order kinetics relate dye removal rates to concentration, providing insights into real-world contact times for target removals. Scalability from 0.01 L to 10 was achieved, with peak flocculation at 10 min for 0.01 L and stabilizes for larger volumes up to 48 h. The *B*. *mycoides* bioflocculant has limited representation in wastewater applications. Collectively, the bridging-driven flocculation and broad operating window indicate a safe, efficient, and thermally tolerant candidate for industrial wastewater treatment with reduced eutrophication risk. The research will progress on alternative, low-cost nutrients for bioflocculant production and on cost analysis as future studies.

## CRediT authorship contribution statement

**Karthikeyan Harinisri:** Writing – original draft, Resources, Methodology, Formal analysis. **Balasubramanian Thamarai Selvi:** Writing – review & editing, Supervision, Conceptualization.

## Declaration of competing interest

The authors declare that they have no known competing financial interests or personal relationships that could have appeared to influence the work reported in this paper.

## Abbreviations used

BLAST: Basic Local Alignment Search Tool
BOD: Biological Oxygen Demand
COD: Chemical Oxygen Demand
CRA: Congo Red Agar
DMEM: Dulbecco's Modified Eagle Medium
DMSO: Dimethyl sulfoxide
DO: Dissolved Oxygen
DPPH: 2,2-Diphenyl-1-picrylhydrazyl
EC: Electrical Conductivity
EPS: Extracellular polymeric substances
FA: Flocculation activity
FBS: Fetal Bovine Serum
FTIR: Fourier Transform Infrared Spectroscopy
MEGA: Molecular Evolutionary Genetics Analysis
MH: Muller-Hinton Broth
min: Minutes
NCBI: National Center for Biotechnology Information
NCCLS: National Committee for Clinical Laboratory Standards
PCR: Polymerase chain reaction
ppm: Parts Per Million
sec: Seconds
SEM: Scanning Electron Microscopy
TDS: Total Dissolved Solids
TSS: Total Suspended Solids
XRD: X-ray diffraction
