# Supplementary material for: Characterization and production of a Bacillus mycoides Bioflocculant for sustainable effluent treatment
Source: Biotechnol Notes. 2026 Feb 7;7:1–15. doi: 10.1016/j.biotno.2026.01.001 (PMC12907645; doi:10.1016/j.biotno.2026.01.001)
Supplement: Multimedia component 1 [file mmc1.docx]

Ethical Clearance Certificate
